# Factors Associated With Adverse Radiographic Outcomes Following Operative Management of Ankle Fractures: A Single-Center Study

**DOI:** 10.7759/cureus.62507

**Published:** 2024-06-17

**Authors:** Ahmed Shirazi, Hasan Alaradi, Hussam A Alanjawi, Ahmed Almeel, Mahmood Alam

**Affiliations:** 1 Orthopaedics and Trauma, Salmaniya Medical Complex, Manama, BHR

**Keywords:** quality of reduction, trimalleolar ankle fracture, bimalleolar ankle fracture, radiographic outcomes, ankle fractures

## Abstract

Introduction

Ankle fractures are commonly encountered fractures in emergency departments worldwide. These fractures often have significant articular involvement that requires anatomic surgical reduction and stabilization. Radiographs can be used in everyday practice to evaluate ankle fractures utilizing various parameters to assess reduction quality intraoperatively and postoperatively.

Several factors have been found to influence the reduction quality of fractures across body regions. This retrospective study aimed to evaluate the influence of several factors on the reduction quality of ankle fractures in a tertiary care center in the Kingdom of Bahrain.

Materials and methods

A total of 462 records were identified during the search, and 68 records were excluded. A total of 394 ankle fractures met the inclusion criteria for the study. Five orthopedic surgeons then evaluated the operative films in accordance with the Delphi consensus parameters for evaluating ankle fractures, and the reductions were graded as good, adequate, or poor.

Results

The study included 394 ankle fractures that met the inclusion criteria, and four significant associations were noted to affect the quality of reduction. Ankle fractures with posterior malleolus involvement (PMI) were significantly associated (p = 0.001) with fragments smaller than 15% and larger than 20% having decreased outcomes. The number of days from admission to operation was also significant (p = 0.009), with the best reductions observed between zero and one day from admission. Operating surgeon was also a significant factor (p = 0.038), with inferior reductions noted in specialist surgeons compared to trainees. The last significant association was the number of malleoli (p = 0.001), with an inferior reduction with a larger number of malleoli involved.

Conclusion

Ankle fractures are a common and significant orthopedic injury. Reduction quality is important for optimal outcomes after surgical stabilization. Various factors including the number of malleoli, the grade of the operating surgeon, and the time of surgery were significantly related to the quality reduction in this single-center study. Expedited surgical management of fractures that are amenable to early fixation, careful assessment, and meticulous technique in fixation of fractures with multiple fractured malleoli is indicated to reduce the chance of malreduction particularly in complex injuries. Further assessment of factors related to reduction quality with large-scale prospective studies would provide orthopedic surgeons with insights into the identification and optimal treatment of such fractures.

## Introduction

Ankle fractures are commonly encountered fractures in emergency departments worldwide, with some authors citing an annual incidence of 187 adults per 100,000 [1]. These fractures often have significant articular involvement that requires anatomical surgical reduction and stabilization [2]. Ankle fractures are regularly managed with open reduction and internal fixation utilizing a variety of orthopedic implants, with the common end goal of achieving the anatomical reduction of the articular surface, appropriate restoration of the anatomic length of the fibula, restoration of the syndesmosis, and preservation of a mechanically stable ankle joint [3]. While reduction and stabilization of ankle fractures are often done by direct visualization of the fracture site, many surgeons use the aid of intraoperative imaging to help confirm restoration of anatomic relations in the ankle mortise.

Several authors found a significant association between functional outcomes, ankle kinematics, and radiological malreduction [4-6]. Anatomic reduction of the ankle mortise has been established as a key factor for the attainment of positive functional outcomes in ankle fractures [4,7]. Radiological outcomes remain the most readily accessible, quantifiable, and reliable parameter that serves to establish if anatomic reduction has been accomplished.

Various parameters have been outlined by several authors to establish the adequacy of reduction in ankle fractures, which include the elimination of articular steps, restoration of the fibular length, reduction of the syndesmosis, and elimination of any talar shift or tilt [8-10]. A Delphi consensus has been recently published in the literature, which aims to streamline the process of radiological evaluation of ankle fractures in everyday practice [11]. This consensus may serve as the new benchmark for orthopedic surgeons in evaluating ankle fractures intraoperatively and postoperatively.

Several factors have been found to influence the reduction quality in fractures across body regions [12,13]. This retrospective study aimed to evaluate the influence of several factors on the reduction quality of ankle fractures in a tertiary care center in the Kingdom of Bahrain.

## Materials and methods

The study was conducted at Salmaniya Medical Complex, a tertiary referral center and the prime trauma center in the Kingdom of Bahrain. A retrospective search was conducted in the operative database, and all records labeled as ankle fractures were retrieved from the operating theater electronic module from January 2019 to August 2023. The study included all ankle fractures (single, two, or three malleoli) in all adult patients. Complex injuries involving the tibial plafond (pilon), revision ankle fractures, pediatric fractures, conservatively managed fractures, fractures with lost surgical documentation, and fractures with no operative radiographs were excluded from the study.

A total of 462 records were identified during the search, and 68 records were excluded. A total of 394 ankle fractures met the inclusion criteria and were included in the study. Table 1 provides a breakdown of all excluded cases by reason for exclusion.

**Table 1:** Reasons for exclusion of record from the study ORIF: Open reduction and internal fixation.

Reasons for exclusion (n = 68)	
Reasons	n (%)
No operative radiographs	6 (8.8)
Pediatric fractures	14 (20.6)
Fractures not involving the ankle joint	20 (29.4)
Duplicate entries	4 (5.9)
Revision surgery	5 (7.4)
No operative notes	5 (7.4)
Not managed with ORIF	1 (1.5)
Wrong identification data	13 (19.1)
Total	68 (100)

Five orthopedic surgeons then evaluated the operative films in accordance with the Delphi consensus parameters for evaluating ankle fractures. All five surgeons underwent a uniform induction process to orient them into a standard application of the Delphi criteria. Two of the surgeons were specialists, two were senior residents, and one evaluator was a consultant foot and ankle surgeon. Poor reduction was defined as the presence of a greater than two-millimeter articular step, intra-articular bone fragments, an articular surface gap greater than two millimeters, restoration of the length of the fibula, and syndesmotic reduction. Adequate reduction was defined as an articular step that is less than two millimeters with a good overall position of the orthopedic implants. A good reduction was an optimal reduction that could not be improved [11].

Data was analyzed using SPSS version 23 (IBM Corp., Armonk, NY) by an independent statistician. This study received approval from the local ethics committee and the research board of the government hospitals (Salmaniya Medical Complex). Qualitative variables were analyzed using the Chi-square test, Fisher’s exact test, and ordinal regression analysis. When qualitative variables were calculated using the Chi-square test and more than 20% of expected values were less than five, Fisher’s exact test was used where feasible.

## Results

Demographic data and characteristics of the dataset

The study included the data of 394 ankle fractures that underwent open reduction and internal fixation between January 2019 and August 2023. A total of 269 (68.3%) participants were male, and 125 (31.7%) were female. The mean age of the sample was 40.9 ± 13.2 years. The majority of the patients included were non-nationals (234, 59.4%) and locals comprised 160 (40.6%). The demographics are detailed in Table 2.

**Table 2:** Frequency and percentage distribution of demographic characteristics of participants

Demographic characteristics	n (%)
Age (n = 394)	
≤30 years	77 (19.5)
31-40 years	141 (35.8)
41-50 years	93 (23.6)
>50 years	83 (21.1)
Median (Q1–Q3)	38 (32–49)
Mean ± SD	40.9 ± 13.2
Gender (n = 394)	
Male	269 (68.3)
Female	125 (31.7)
Nationality (n = 394)	
Bahraini	160 (40.6)
Non-Bahraini	234 (59.4)

Of the 394 total fractures included in the study, 169 (42.9%) involved a single malleolus, 159 (40.4%) involved two malleoli, and 66 (16.8%) involved three malleoli. Of the total included fractures, 201 (51.0%) were classified according to the Lauge-Hansen (LH) classification as follows: 75 (37.3%) had supination-external rotation (SER), 61 (30.3%) had pronation-external rotation (PER), 47 (23.4%) had pronation-abduction (PAB), and 18 (9%) had supination-adduction (SAD).

Ankle fractures with medial malleolus (MM) involvement, which were nonclassifiable with the LH classification, were categorized by fracture pattern description. MM involvement was present in 72 (18.3%) of the sample size and was classified as follows: 35 (48.6%) had transverse patterns, 24 (33.3%) had oblique patterns, and 13 (18.1%) had vertical patterns.

Similarly, ankle fractures with lateral malleolus (LM) involvement, which were nonclassifiable with the LH classification, were classified by the Weber classification. A total of 120 fractures fit the definition and were classified according to the Weber classification: The sample had 85 (70.8%) Weber B fractures, 32 (26.7%) Weber C fractures, and three (2.5%) Weber A fractures.

Posterior malleolus involvement (PMI) was measured as a percentage of involvement of the tibial plafond in all the included ankle fractures. PMI was recorded as follows: 39 (42.9%) had 15%-20% involvement, 35 (38.5%) had less than 15% involvement, and 17 (18.7%) had more than 20% involvement.

Laterality was recorded, and 203 (51.5%) had right ankle fractures, while 191 (48.5%) had left ankle fractures. Days to operation were grouped as follows: 198 (50.3%) underwent surgery on the day of admission or day one, 93 (23.6%) underwent surgery on days two or three, and 103 (26.1%) underwent surgery on day four or beyond. Diabetes mellitus was noted in 41 (10.4%), while 353 (89.6%) had no established diagnosis of diabetes. Other comorbidities were present in 70 (17.8%), while 324 (82.2%) had no documented pre-existing medical conditions. Only 62 (15.7%) had multiple injuries (defined as injuries to multiple limbs or multiple organ systems), and 332 (84.3%) had isolated ankle fractures. The timing of surgery was registered by shift; 194 (49.2%) underwent surgical intervention during the morning shift, 195 (49.5%) during the evening shift, and five (1.3%) during the night shift. The grade of the operating surgeon was grouped into either trainee or specialist. Trainees operated 225 (57.1%) cases, while specialists operated 169 (42.9%) cases.

The quality of reduction was grouped into three distinct groups. Good reduction was observed in 243 (61.7%), adequate reduction in 112 (28.4%), and poor reduction in 39 (9.9%). Suboptimal reductions, including all adequate and poorly graded reductions, were observed in 151 ankle fractures (38.3), and the reason for the suboptimal reduction was collected. An articular step accounted for 98 (64.9%) suboptimal reductions, intra-articular hardware in 33 (21.9%), syndesmotic malreduction in 18 (11.9%), and fibular shortening in two (1.3%). The fracture characteristics are included in Table 3.

**Table 3:** Frequency and percentage distribution of ankle fracture characteristics of participants SER: Supination-external rotation; PAB: Pronation-abduction; PER: Pronation-external rotation; SAD: Supination-adduction.

Ankle fracture characteristics	n (%)
Number of fractured malleoli (n = 394)	
One	169 (42.9)
Two	159 (40.4)
Three	66 (16.8)
Lauge-Hansen classification (n = 201)	
SER	75 (37.3)
PAB	47 (23.4)
PER	61 (30.3)
SAD	18 (9)
Medial malleolus (not fitting Lauge-Hansen classification) (n = 72)	
Transverse	35 (48.6)
Vertical	13 (18.1)
Oblique	24 (33.3)
Weber classification (other than Lauge-Hansen classification) (n = 120)	
A	3 (2.5)
B	85 (70.8)
C	32 (26.7)
Posterior malleolus involvement (n = 91)	
<15%	35 (38.5)
15-20%	39 (42.9)
>20%	17 (18.7)
Median (Q1–Q3)	20 (10–20)
Mean ± SD	17.2 ± 8.6
Side affected (n = 394)	
Right	203 (51.5)
Left	191 (48.5)
Number of days to operation (n = 394)	
0-1	198 (50.3)
2-3	93 (23.6)
>3	103 (26.1)
Median (Q1–Q3)	1 (1–4)
Mean ± SD	2.7 ± 3.1
Presence of diabetes (n = 394)	
Yes	41 (10.4)
No	353 (89.6)
Presence of other comorbidities (n = 394)	
Yes	70 (17.8)
No	324 (82.2)
Grade of operating surgeon (n = 394)	
Specialist	169 (42.9)
Trainee	225 (57.1)
Presence of other injuries (n = 394)	
Yes	62 (15.7)
No	332 (84.3)
Time of surgery (n = 394)	
Morning	194 (49.2)
Evening	195 (49.5)
Night	5 (1.3)
Quality of reduction according to Delphi Consensus (n = 394)	
Good	243 (61.7)
Adequate	112 (28.4)
Poor	39 (9.9)
Reason for suboptimally graded reduction (n = 151)	
Articular step	98 (64.9)
Intra-articular hardware	33 (21.9)
Syndesmotic malreduction	18 (11.9)
Fibular shortening	2 (1.3)

Association between demographics and fracture characteristics and quality of reduction

Analysis showed no significant association between age, gender, or nationality and the quality of reduction. Analysis of fracture characteristics and patterns revealed several significant associations. The number of fractured malleoli was significantly associated with inferior quality of reduction (p = 0.001), with multiple fractured malleoli having an increased risk of suboptimal reduction. Single malleolus fractures had the highest number of good reductions (122, 72.2%), while only 30 (45.5%) of trimalleolar fractures had good reductions. When comparing suboptimal reductions, single malleolus fractures had poor reductions in 13 cases (7.7%), bimalleolar in 20 cases (12.6%), and trimalleolar in six cases (9.1%).

PMI was significantly associated with inferior reduction quality (p = 0.001). The data showed an increased risk of suboptimal reduction in both extremes. When PMI was less than 15%, adequate reduction was noted in 17 (48.6%) cases and poor reduction in one case (2.9%). Similarly on the other end of the spectrum, when PMI was more than 20%, adequate reduction was noted in four cases (23.5%) and poor reduction in seven cases (41.2%). When PMI was within the range of 15%-20%, good reduction was measured in 25 (64.1%) cases, adequate reduction in 13 cases (33.3%), and poor reduction in one case (2.6%).

Days to the operation date were found to be significantly associated with an increased risk of suboptimal reduction quality (p = 0.009). Fractures operated on the day of admission or day one had good reductions in 139 (70.2%) cases and poor reductions in 14 (7.1%). Fractures operated on days two and three had good reductions in 49 (52.7%) cases. Fractures operated after three days had good reductions in 55 (53.4%) cases and poor reductions in 15 (14.6%) cases.

The last significant association was between the grade of the operating surgeon and inferior reduction quality (p = 0.038). Specialists had good reductions in 92 (54.5%) cases, while trainees had 151 (67.1%) cases with good reduction. Specialists had higher suboptimal outcomes, with adequate reductions noted in 57 cases (33.7%) and poor reductions in 20 cases (11.8%). Trainees had lower numbers of suboptimal outcomes, with adequate reductions in 55 cases (24.4%), and poor reductions in 19 cases (8.4%).

All other fracture characteristics showed no significant associations. The associations between quality of reduction, demographics, and fracture characteristics are outlined in Tables 4, 5.

**Table 4:** Association between the quality of reduction and demographic characteristics of participants ^a ^P-value was calculated by the Chi-square test.

Demographic characteristics	Quality of reduction			Chi-square value	P-value
	Good	Adequate	Poor		
	n (%)	n (%)	n (%)		
Age					
≤30 years	50 (64.9)	18 (23.4)	9 (11.7)	3.719	0.715^a^
31-40 years	84 (59.6)	43 (30.5)	14 (9.9)		
41-50 years	58 (62.4)	24 (25.8)	11 (11.8)		
>50 years	51 (61.4)	27 (32.5)	5 (6)		
Gender					
Male	165 (61.3)	74 (27.5)	30 (11.2)	1.613	0.446^a^
Female	78 (62.4)	38 (30.4)	9 (7.2)		
Nationality					
Bahraini	102 (63.8)	43 (26.9)	15 (9.4)	0.491	0.782^a^
Non-Bahraini	141 (60.3)	69 (29.5)	24 (10.3)		

**Table 5:** Association between the quality of reduction and fracture characteristics of participants ^a^ P-value was calculated by the Chi-square test ^b^ P-value was calculated by the Fisher’s exact test. SER: Supination-external rotation; PAB: Pronation-abduction; PER: Pronation-external rotation; SAD: Supination-adduction.

Ankle fracture characteristics	Quality of reduction			Chi-square or Fisher’s value	P-value
	Good	Adequate	Poor		
	n (%)	n (%)	n (%)		
Number of fractured malleoli					
One	122 (72.2)	34 (20.1)	13 (7.7)	19.391	0.001^a^
Two	91 (57.2)	48 (30.2)	20 (12.6)		
Three	30 (45.5)	30 (45.5)	6 (9.1)		
Lauge-Hansen classification					
SER	37 (49.3)	30 (40)	8 (10.7)	7.737	0.258^a^
PAB	28 (59.6)	12 (25.5)	7 (14.9)		
PER	33 (54.1)	24 (39.3)	4 (6.6)		
SAD	6 (33.3)	8 (44.4)	4 (22.2)		
Medial malleolus (not fitting Lauge-Hansen classification)					
Transverse	28 (80)	5 (14.3)	2 (5.7)	3.213	0.547^b^
Vertical	10 (76.9)	3 (23.1)	0 (0)		
Oblique	17 (70.8)	7 (29.2)	0 (0)		
Weber Classification (other than Lauge-Hansen classification)					
A	3 (100)	0 (0)	0 (0)	4.543	0.281^b^
B	62 (72.9)	13 (15.3)	10 (11.8)		
C	18 (56.3)	10 (31.3)	4 (12.5)		
Posterior malleolus involvement					
<15%	17 (48.6)	17 (48.6)	1 (2.9)	17.806	0.001^b^
15%-20%	25 (64.1)	13 (33.3)	1 (2.6)		
>20%	6 (35.3)	4 (23.5)	7 (41.2)		
Side affected					
Right	132 (65)	55 (27.1)	16 (7.9)	2.744	0.254^a^
Left	111 (58.1)	57 (29.8)	23 (12)		
Number of days to operation					
0-1	139 (70.2)	45 (22.7)	14 (7.1)	13.527	0.009^a^
2-3	49 (52.7)	34 (36.6)	10 (10.8)		
>3	55 (53.4)	33 (32)	15 (14.6)		
Presence of diabetes					
Yes	23 (56.1)	16 (39)	2 (4.9)	3.204	0.201^a^
No	220 (62.3)	96 (27.2)	37 (10.5)		
Presence of other comorbidities					
Yes	46 (65.7)	18 (25.7)	6 (8.6)	0.597	0.742^a^
No	197 (60.8)	94 (29)	33 (10.2)		
Grade of operating surgeon					
Specialist	92 (54.4)	57 (33.7)	20 (11.8)	6.560	0.038^a^
Trainee	151 (67.1)	55 (24.4)	19 (8.4)		
Presence of other injuries					
Yes	45 (72.6)	14 (22.6)	3 (4.8)	4.206	0.122^a^
No	198 (59.6)	98 (29.5)	36 (10.8)		
Time of surgery					
Morning	124 (63.9)	55 (28.4)	15 (7.7)	3.142	0.488^b^
Evening	116 (59.5)	56 (28.7)	23 (11.8)		
Night	3 (60)	1 (20)	1 (20)		

Ordinal regression analysis

Factors that were found to be significant in univariate analysis were chosen for further assessment utilizing an ordinal regression model. One of the variables (days to operation date) was numeric; therefore, it was entered into the ordinal regression model as a continuous variable, which was not significant. However, upon grouping the variables, the results were significant. Both analyses are included in Tables 6, 7. The analysis was then repeated, with the variable segmented into categories to determine significance.

**Table 6:** Ordinal regression between the independent variables and quality of reduction, with days as a continuous variable The dependent variable is the quality of reduction.

Independent variables	Estimate	P-value	95% CI	
			Lower	Upper
Number of fractured malleoli	-0.435	0.003	-0.718	-0.153
Number of days to operation	-0.053	0.098	-0.115	0.010
Grade of operating surgeon	-0.464	0.026	-0.873	-0.055

**Table 7:** Ordinal regression between the independent variables and quality of reduction, with segmented days to operation The dependent variable is the quality of reduction.

Independent variables	Estimate	P-value	95% CI	
			Lower	Upper
Number of fractured malleoli	-0.415	0.004	-0.696	-0.133
Number of days to operation	-0.346	0.005	-0.587	-0.104
Grade of operating surgeon	-0.475	0.023	-0.886	-0.065

All three variables showed a statistically significant association with the quality of reduction. The number of fractured malleoli had an estimate of -0.415 (CI = -0.696, -0.133) (p = 0.004). The number of days to the operation date had an estimate of -0.346 (CI = -0.587, -0.104) (p = 0.005). The grade of the operating surgeon had an estimate of -0.475 (CI = -0.886, -0.065) (p = 0.023).

## Discussion

Ankle fractures are a commonly encountered fracture with a high annual incidence among the population, which is ever-increasing due to the aging population [14,15]. Management of such fractures frequently entails surgical management; therefore, the meticulous treatment and optimal reduction of these fractures are crucial for ideal outcomes [16,17]. We found that a tibiotalar shift of one millimeter of lateral displacement can cause a reduction of up to 42% in the contact area, which has been hypothesized to lead to post-traumatic arthritis [6]. The authors have also noted in the literature that the malreduction of the fibula, caused by rotational malalignment or shortening, is associated with decreased contact area [18]. We found that the syndesmotic injury was also important in the onset of ankle osteoarthritis [19]. Malreduction and decreased contact area have been suggested to lead to post-traumatic osteoarthritis and decreased quality of life [20,21].

Radiological parameters were used to assess reduction quality in our study, as outlined in the Delphi consensus [11]. Although many methods exist to assess reduction, no unified method is universally accepted in the literature. The Delphi consensus suggested a simplified and streamlined approach to assess the reduction to allow observers to accurately determine the reduction quality. Several published articles acknowledge the importance of anatomical reduction on outcomes [4,7,22].

The number of fractured malleoli was significantly associated with reduction quality, with the expected result of trimalleolar fractures having higher rates of poor and adequate reductions when compared to single malleolus and bimalleolar fractures. This is likely reflective of the relationship between multiple malleolar fractures with higher energy trauma and more complex fracture patterns, resulting in more difficult reductions [23].

The findings in our study highlight several factors that are significantly associated with the reduction quality in our institution. Posterior malleolus fracture size was found to be significantly associated with poor reduction quality on either end of the size spectrum; larger fragments of more than 20% of the plafond and smaller fragments of less than 15% were associated with poor reduction quality in comparison to fragments between 15% and 20% of the tibial plafond. This highlights the fact that the size of the fragment does not directly contribute to reduction quality, but the pattern of the fracture and potential comminution likely play a more significant role that cannot be simply accounted for by measuring the percentage involvement of the articular surface [22]. Further studies should aim to draw more specific characterizations of posterior malleolus fragments that could account for pattern, comminution, and anatomical location to better understand the factors leading to malreduction [24].

Another finding of note in our study is the apparent paradoxical relationship between training level and quality of reduction. Trainees were found to have significantly higher proportions of good and adequate reductions when compared to their specialist counterparts. While this finding may seem counterintuitive, more complex fracture patterns are likely assigned to more senior team members, while simpler fracture patterns are assigned to trainees. This discrepancy in fracture complexity was not adjusted for in our study and remains an important confounder in the interpretation of the significance of this association.

The time to surgical intervention from admission was found to be significantly associated with reduction quality; this finding is in line with the findings of various studies that established the importance of early reduction and fixation in various fractures across the body [25,26]. Our study found that early surgical intervention (between zero and one day from admission) was significantly associated with a better quality of reduction. This result should be interpreted with caution as many confounders could explain this result. High-energy trauma and more complex injuries likely go in line with significant soft tissue compromise, which necessitates a delay in surgical intervention, while inversely low-energy trauma and therefore simpler fracture patterns are less likely to injure the soft tissue envelope to the same extent. Surgeons should exercise their judgment in assessing soft tissue compromise and further damage to the envelope while balancing the potential of early intervention on improved reduction quality. Further studies aiming to determine the factors related to malreduction should consider the evaluation of the mechanism of injury, soft tissue status, and fracture complexity. The retrospective nature of this study significantly limited the evaluation of these confounders.

The authors acknowledge that the study has several limitations. The retrospective nature of this study has limited access to several confounders that may have a bearing on the interpretation of the results. While authors have sought to devise a unified process for the assessment of radiographs, inter-observer variance is inevitable and may contribute to some of the findings in the study. Further, while radiographs are a cost-effective and accessible tool in the assessment and classification of ankle fractures, they may not be as accurate as other modalities such as CT or MRI assessment. Some ankle fractures identified as good or adequate may be poor by virtue of syndesmotic malreduction or articular steps that can only be confirmed with advanced imaging technologies. Finally, this was a single-center study with a good but limited sample size, which may affect the generalizability of the findings and the interpretation of the data.

## Conclusions

Ankle fractures are a common and important orthopedic injury. Reduction quality is important for optimal outcomes after surgical stabilization. Various factors including the number of malleoli, the grade of the operating surgeon, and the time of surgical intervention were found to be significantly related to the reduction quality in this single-center study. Expedited surgical management of fractures that are amenable to early fixation, careful assessment, and meticulous technique in fixation in fractures with multiple fractured malleoli is indicated to reduce the chance of malreduction particularly in complex injuries. Further assessment of factors related to reduction quality with large-scale prospective studies would provide orthopedic surgeons with insights into the identification and optimal treatment of such fractures.
